# “We might get a lot more families who will agree”: Muslim and Jewish perspectives on less invasive perinatal and paediatric autopsy

**DOI:** 10.1371/journal.pone.0202023

**Published:** 2018-08-09

**Authors:** Celine Lewis, Zahira Latif, Melissa Hill, Megan Riddington, Monica Lakhanpaul, Owen J. Arthurs, John C. Hutchinson, Lyn S. Chitty, Neil J. Sebire

**Affiliations:** 1 North East Thames Regional Genetics Service, Great Ormond Street Hospital for Children NHS Foundation Trust, London, United Kingdom; 2 Genetics and Genomic Medicine, The UCL Great Ormond Street Institute of Child Health, London, United Kingdom; 3 College of Medical and Dental Sciences, University of Birmingham, Birmingham, United Kingdom; 4 School of Medicine, Faculty of Medicine and Health Sciences, University of Nottingham, Nottingham, United Kingdom; 5 Department of Psychological Services, Great Ormond Street Hospital for Children NHS Foundation Trust, London, United Kingdom; 6 Faculty of Population Health Sciences, UCL Great Ormond Street Institute of Child Health, London, United Kingdom; 7 Community Paediatrics, Whittington Health NHS Trust, London, United Kingdom; 8 Department of Radiology, Great Ormond Street Hospital for Children NHS Foundation Trust, London, United Kingdom; 9 Department of Histopathology, Great Ormond Street Hospital for Children NHS Foundation Trust, London, United Kingdom; Emory University, School of Public Health, UNITED STATES

## Abstract

**Background:**

Perinatal and paediatric autopsy rates are at historically low levels with declining uptake due to dislike of the invasiveness of the procedure, and religious objections particularly amongst Muslim and Jewish parents. Less invasive methods of autopsy including imaging with and without tissue sampling have been shown to be feasible alternatives. We sought to investigate attitudes including religious permissibility and potential uptake amongst members of the Muslim and Jewish communities in the United Kingdom.

**Methods:**

Semi-structured interviews with religious and faith-based authorities (n = 16) and bereaved parents from the Jewish community (n = 3) as well as 10 focus groups with community members (60 Muslim participants and 16 Jewish participants) were conducted. Data were analysed using thematic analysis to identify key themes.

**Findings:**

Muslim and Jewish religious and faith-based authorities agreed that non-invasive autopsy with imaging was religiously permissible because it did not require incisions or interference with the body. A minimally invasive approach was less acceptable as it still required incisions to the body, although in those circumstances where it was required by law it was more acceptable than a full autopsy. During focus group discussions with community members, the majority of participants indicated they would potentially consent to a non-invasive autopsy if the body could be returned for burial within 24 hours, or if a family had experienced multiple fetal/pregnancy losses and the information gained might be useful in future pregnancies. Minimally invasive autopsy was less acceptable but around half of participants might consent if a non-invasive autopsy was not suitable, with the exception of the Jewish Haredi community who unanimously stated they would decline this alternative.

**Conclusions:**

Our research suggests less invasive autopsy offers a viable alternative to many Muslim and Jewish parents in the UK who currently decline a full autopsy. The findings may be of importance to other countries with significant Muslim and/or Jewish communities as well as to other religious communities where concerns around autopsy exist. Awareness-raising amongst religious leaders and community members will be important if these methods become routinely available.

## Introduction

Although there is good evidence that autopsy is the single most useful investigation in providing information on cause of death [1], consent to this in the perinatal and paediatric setting is declining across the developed world [2–5]. Fewer than half of parents of stillborn babies and only a quarter of parents of neonate deaths consent for traditional autopsy in the United Kingdom (UK) [6]. Reasons for this include dislike of the invasive procedure, fear of disfigurement, ambivalence about the value of an autopsy, and, particularly amongst Muslim and Jewish parents, religious objections [7–9].

There are 2.7 million Muslims currently living in England and Wales, and 263,000 Jews, with both populations growing [10]. Moreover, two-thirds of Muslims are of Asian origin (primarily Pakistan and Bangladesh), and they have the second highest risk for stillbirth (6.3 per 1,000 births compared to 4.16 per 1,000 for UK population), neonatal death (2.3 per 1,000 birth compared to 1.77 per 1,000 for UK population) and extended perinatal death (8.7 per 1,000 births compared to 5.92 per 1,000 for UK population) in the United Kingdom [6]. For both Islam and Judaism, cutting and disfigurement of the deceased, and removing internal organs, tissue and fluids, is forbidden, and there is a religious requirement to bury the body as soon as possible [11–14]. Thus traditional autopsy is not permitted unless it is required by law or would directly save another life [13, 15, 16]. Autopsy might also be permitted by a religious authority if a miscarriage has occurred early in pregnancy before the soul is thought to have emerged in the fetus, [17] if it might provide valuable information about risk for future pregnancies [18] or for other compelling reasons including benefit to society or prevention of disease [19]. Empirical evidence has highlighted uptake of fetal or neonatal autopsy amongst Muslim parents of 23% [20] and 42% [21] compared 77% [20] and 75% [21] amongst non-Muslim parents, although the specific circumstances of those Muslim parents who consented is not clear.

Recently a number of less invasive approaches to autopsy have been developed. Non-invasive autopsy (NIA) does not require body incisions, but uses post-mortem cross-sectional imaging techniques such as computed tomography (CT) or magnetic resonance imaging (MRI) with microbiology and placental examination where appropriate. It has been shown to be highly accurate for the detection of congenital anatomical abnormalities, such as intracranial haemorrhage, brain malformations, renal anomalies, congenital heart disease and skeletal dysplasias [22].Minimally invasive autopsy (MIA), which includes post-mortem imaging as well as laparoscopic or image-guided tissue sampling requiring only a small incision, has also been shown to be feasible and effective with tissue biopsy providing additional diagnostic information to imaging alone in the majority of cases. [23] This approach is not yet in widespread clinical use in developed countries [23] but needle-based sampling is increasingly being used in sub-Saharan Africa and south Asia where rates of childhood mortality are high [24].

Either or both of these approaches to autopsy may offer an acceptable alternative to Muslim and Jewish parents. A study conducted in Belgium indicated that 65% of Muslim participants would hypothetically consent for a minimally invasive procedure, however, the sample size was small (n = 13) and non-invasive autopsy was not included as an alternate method [21]. A study conducted in Africa and South Asia on hypothetical willingness to consent to MIA (defined as a targeted small needle biopsy) found acceptance rates of around 70% amongst Muslim relatives of deceased individuals[25]. Such findings may not be applicable in the UK where autopsy is more frequently conducted (it is a legal requirement in certain circumstances) and where a different healthcare system exists. Further research is required to assess the acceptability of these methods. In 2016 the authors of this paper received an NIHR Health Technology Assessment (HTA) award to address the question “what is the acceptability of less invasive autopsy (MIA and NIA) amongst key stakeholders?” As part of this study we conducted a qualitative study comprising focus groups and interviews with members of the Muslim and Jewish faiths (conducted between April 2016 and May 2017). The objectives were to 1) identify whether NIA and/or MIA were religiously permissible methods of autopsy, and 2) examine whether the availability of these less invasive methods would increase uptake from within those communities.

## Methods

### Ethical approval and consent to participate

Ethical approval for this study was obtained through London-Bloomsbury Research Ethics Committee (16/LO/0248). All participants provided written informed consent.

### Study setting

This study was conducted in the UK. Interview participants (bereaved parents and religious leaders) were based in London and the Midlands. Focus groups with the Muslim community were conducted in either Tower Hamlets, East London or Stoneygate, Leicester. Leicester is one of the most ethnically and culturally diverse cities in the UK with 37% of residents classifying their ethnicity as Asian or Asian British (28% as Indian, 2% Pakistani and 1% Bangladeshi) and 19% classifying their religion as Muslim [26]. In Stoneygate, 44% of the population are economically active (employed full-time, part-time, or self-employed), 7% are unemployed, 8% are retired, 17% are students and 12% are either looking after home/family or are long-term sick or disabled.[27] In the London borough of Tower Hamlets, 41% of the population classify themselves as Asian or Asian British (32% as Bangladeshi, 3% as Indian and 1% as Pakistani)[28] and 38% classify their religion as Muslim [29] In the Spitalfields and Banglatown ward in Tower Hamlets where the focus groups took place, 55% of the population are economically active, 7% are unemployed, 4% are retired, 13% are students and 10% are either looking after home/family or are long-term sick or disabled.[27]

Focus groups with the Jewish community were conducted in Golders Green and Stamford Hill, both in North London. London has the UK’s largest Jewish community [30]. Around 50% of the 300,000 or so Jews that live in the UK are affiliated with Orthodox Judaism and 13% with Strictly Orthodox (Haredi). [31] In Golders Green ward, 61% of the population are economically active, 5% are unemployed, 7% are retired, 9% are students and 11% are either looking after home/family or are long-term sick or disabled.[27] In Springfield ward, Stamford Hill, 50% of the population are economically active, 8% are unemployed, 6% are retired, 11% are students and 16% are either looking after home/family or are long-term sick or disabled.[27]

### Study design

This was a qualitative study comprising standard data collection approaches, namely semi-structured interviews (with bereaved parents, religious leaders and faith based authorities and focus groups (with members of the Muslim and Jewish community). [32] The methodological approach taken to recruit participants followed recognised approaches for working with minority ethnic communities in the UK. [33, 34]

### Recruitment

#### Interviews with religious and faith-based authorities

Interviews were conducted either face-to-face (at the workplace of the interviewee) or by telephone. CL conducted interviews with religious leaders/faith-based advocates and either CL or MR conducted interviews with bereaved parents. A Muslim chaplain and an Orthodox Rabbi based in London with links to a participating hospital were identified as key informants. They were initially contacted by CL via email with further face-to-face follow up to discuss recruiting additional participants. Those key informants then suggested other religious leaders (Rabbi or Imam) and faith-based authorities (e.g. hospital chaplain, religious scholar, spokesperson for the Muslim burial society) for CL to invite into the study. In some cases, interviewees suggested other religious or faith-based authorities that might have an interest in the study (a ‘snowballing’ approach). Purposive sampling was used throughout whereby CL selected potential participants with diverse backgrounds (e.g. health advocate, spokesperson for Jewish Medical Association) beliefs and practices who spoke English in order to maximise variation within the sample. For example, to explore potentially diverse viewpoints amongst the Jewish religious leaders we included Rabbis from within the Haredi, Orthodox, Masorti and Liberal movements who have differing beliefs and practices. Where two or three religious leaders from within one movement were suggested we initially only selected one of those to invite for an interview. Interviews with religious leaders from other faiths (Protestant Christian, Catholic Christian, Hindu) were also included in order to explore how the Muslim and Jewish viewpoint differed from that of other dominant faiths in the UK. Potential participants were sent an email inviting them to take part in the study along with a participant information sheet. Those interested in taking part were asked to respond to the researcher. Interviews with religious and faith-based authorities took place between April and July 2016.

#### Community focus groups

Focus groups with members of two Muslim communities were arranged through representatives from the East Midlands Centre for Black Minority Ethnic (BME) Health in Leicester and a Muslim community centre in Tower Hamlets, East London. At both sites the recruiter was a member of the Muslim community. For the Jewish community these were arranged through a Rabbi from the Orthodox community and a faith-based authority with close links to the Haredi community. Purposive sampling was used to recruit parents (male and female) who were of child-bearing age (18–50 years). For the Muslim focus groups, we ensured the two main South Asian ethnic groups in the UK (Pakistani and Bangladeshi) were represented [10]. Participants who spoke English, Urdu or Bengali were included.

Recruitment occurred in a number of ways: direct contact with participants attending community centres to discuss the study and invite them to take part (Bangladeshi and Pakistani community in Leicester), approaching community members by telephone (Haredi community in London and members of the Muslim community in East London), approaches at coffee mornings (Muslim community in East London), asking female participants to invite their husbands to take part (Bangladeshi and Pakistani Muslim community in Leicester) and circulating an email to members of a synagogue (Orthodox Jewish community in London). Interested participants were asked to reply to the community representative (for the Muslim groups), CL directly (for the Jewish Orthodox group) or the faith-based authority (for the Jewish Haredi group). Focus groups took place between September 2016 and May 2017.

#### Interviews with bereaved parents

Muslim or Jewish parents who had experienced bereavement (termination of pregnancy for fetal abnormality, stillbirth, neonatal or child death) were recruited either retrospectively through one of four support groups (Antenatal Results and Choices, Sands, The Lullaby Trust and Child Bereavement UK) or prospectively through one of six hospital sites located in London and regional England to complete a survey to assess acceptability and likely uptake (paper in progress). At the end of the survey responders were asked to leave their contact details if they were willing to take part in a telephone interview to discuss their views about autopsy in more depth. No time-limit was set in terms of how many months prior to the interview the loss occurred. Two of the hospitals were specifically selected because their catchment area included a significant Muslim and Jewish population base. Interviews took place between December 2016 and January 2017.

### Data collection

Data were collected by a female social scientist with experience in conducting qualitative studies (CL) and a female clinical psychologist with a PhD which involved conducting and analysing qualitative interviews (MR).

Informed by the authors’ previous work [7], separate but related topic guides (S1–S3 Appendices) were developed for the interviews and focus groups in collaboration with the advisory team comprising a pathologist, a radiologist, a fetal medicine consultant, an anatomical pathology technologist, a social scientist and patient support advocates. The following topic areas were included: participants personal views regarding acceptability of traditional autopsy examination and when, if at all, it would be acceptable; acceptability of traditional autopsy from a Muslim/Jewish perspective; personal views regarding NIA and MIA; permissibility of NIA and MIA from a Muslim/Jewish perspective (both religious belief and practice); likely uptake of NIA and MIA both personally and within the community more generally; recommendations for improving uptake of NIA and MIA within the community.

During interviews and focus groups, participants were given a description of a full autopsy, NIA (imaging using either an MRI or CT scan and may also include examination of the placenta or bloods) and MIA (imaging alongside examination of internal organs and tissue sampling done using a ‘keyhole surgery’ approach), which were developed by CL and NJS and reviewed by the advisory team to ensure they were clear, accurate and worded sensitively (S4 Appendix). Focus group participants were also shown an image of an MRI machine and laparoscopic equipment to enhance understanding of NIA and MIA. We did not provide information upfront about how long it would take to conduct the procedure and return the body for burial as in practice this will be dependent on circumstances such as which hospital the body is located, the availability of scanning equipment, the availability of the radiologist or pathologist etc. However, we did discuss with participants how long they would be prepared to wait to have a less invasive procedure.

Participants were interviewed by either CL or MR and the interview was conducted either face-to-face or over the telephone. Focus groups with Muslim participants were conducted at Muslim community centres in Tower Hamlets and Leicester and focus groups with Jewish participants were conducted at the home of a Rabbi and the home of a faith-based authority. Where advised, focus groups were divided by gender to take account of religious or cultural requirements. Focus groups were facilitated by CL and a translator from within the community who was present at groups where English language was a potential barrier.

Written consent was sought to record the interviews and focus groups discussions and use anonymised quotes in papers or reports. When non-English speaking participants were present at focus groups, a translator began by translating the participant information sheet and consent form into Urdu or Bengali. During these focus groups, there was two-way translation between the facilitator and participants who did not speak English. All interviews and focus groups were recorded using a digital voice recorder and transcribed verbatim. A translator transcribed those recordings where participants spoke in Urdu and Bengali, and translated those sentences into English. No observer was present at the focus group discussions, however CL noted general reflections on the group discussion immediately after the session had ended. Both interview and focus group participants received a gift voucher as appreciation for their time and contribution.

### Data analysis and validation

Analysis was facilitated using Nvivo version 10 (QSR International, Pty Ltd) software. Thematic analysis was used to summarise respondents’ views [35]. Data were collected and analysed concurrently with data collection ceasing once saturation had been reached. The three bereaved parent interviews included in the data analysis supported the findings from the parent focus groups as no significant codes or new themes were derived when analysing these transcripts. Focus groups and interviews were coded as one data set. Four researchers were involved in the coding process. A subset of transcripts were independently coded by at least two researchers using separate Nvivo files which were then merged to compare codes. Once a coding framework had been agreed, the remaining transcripts were independently coded according to the framework. Codes were then clustered to form overarching themes. The majority of the overarching themes were closely aligned with the semi-structured topic guide, however code names were derived inductively from the data. In the final stages of data analysis, findings were discussed with key informants from both faiths (member checking) to check the validity of the interpretation of the data [36]. This involved showing the draft manuscript to key informants; checking that the themes were reflective of the community viewpoints (i.e. asking “do the themes cited here reflect how you think the community feels about this topic?”, and ensuring the discussion and recommendations for practice were appropriate.

## Results

### Participants

Sixteen of 19 (84%) religious or faith-based authority who were approached for interview participated (Table 1). Eight focus groups comprising 60 Muslim participants and two with 16 Jewish participants were conducted (Table 2). Interviews lasted between 16 minutes and one hour (average 36 minutes); focus groups lasted 1.5 to 2 hours and consisted of between 6 and 10 participants per group.

**Table 1:** Religious and faith-based authority participants.

Religious or faith-based authority	N = 16
**Muslim**	n = 6
Muslim Chaplain	3
Imam	1
Spokesperson for Muslim Cemetery Trust	1
Scholar in Islamic Bioethics	1
**Jewish**	n = 6
Rabbi	3
Spokesperson for Jewish Medical Association	1
Spokesperson for Jewish Burial Society	1
Orthodox Jewish Health Advocate	1
**Christian**	n = 3
Church of England Chaplain	1
Anglo Catholic Chaplain	1
Roman Catholic Chaplain	1
Hindu	n = 1
Hindu Chaplain	1

**Table 2:** Focus group participant characteristics.

	**Total N = 76**	**Muslim n = 60**	**Jewish n = 16**
**Age Range [Mean]**	19–49 [38.05]	19–49 [38]	29–49 [37]
Gender			
Male	25	20	5
Female	51	40	11
**Religion**			
Muslim	60	/	/
Jewish	17	/	/
**Religiosity**			
Very Religious	23	12	11
Quite religious	45	43	2
Not Very Religious	5	3	2
**Country of Birth**			
United Kingdom	24	11	13
Bangladesh	24	24	0
Pakistan	16	16	0
Somalia	5	1	0
Philippines	1	5	0
Thailand	1	1	0
Morocco	1	1	0
Indonesia	1	1	0
Belgium	1	0	1
South Africa	1	0	1
USA	1	0	1
**Education**			
No formal qualification	11	9	2
GSCE or equivalent	19	14	5
A-level or equivalent	14	14	0
Degree or equivalent	17	16	1
Postgraduate Degree	14	6	8
**Experience of loss**			
Miscarriage (<12 wks)	26	21	5
Miscarriage (12–24 wks)	8	7	1
Stillbirth	4	2	2
Termination for fetal anomaly	2	1	1
Neonatal/infant death (0–12 mos)	2	1	1
Child death (1–16 yrs)	3	1	2
**If YES, were you approached about autopsy in any of those cases?**			
Yes	13	9	4
No	15	12	3
Not sure	3	3	0
**If YES, did you consent?**			
Yes	5	4	1
No	8	5	3

Six Muslim and six Jewish participants completed the survey, however only three Jewish survey responders indicated their willingness to take part in telephone interviews (all recruited retrospectively through a support group) which were conducted between December 2016 and January 2017 (all three were female; age range 50–64; all educated to A level or above; two self-reported as being quite religious, one self-reported as being not very religious). One had experienced a neonatal/infant death and had a Coronial autopsy, one had a stillbirth and one a termination for a fetal anomaly; both had consented to a full autopsy. None of the survey responders from the Muslim community (n = 6) agreed to be contacted further.

### Key themes

Participants acknowledged that religious devotion involved a negotiation between religious observance and the complexities of daily life. For some participants, it was not always possible to *“live our lives as strictly as we would like”* (FG9, Pakistani Muslim, Women) or that sometimes, for example when a tragedy occurs, one’s emotions overrode one’s observance of religious beliefs and practices.

*“Everyone’s faith goes up and down*, *so when the tragedy does happen to you*, *it depends what emotional state you're at*, *a lot of things comes into it*, *sometimes instead of religion coming into it*, *it’s more your personal feelings*.*”* FG7, Mixed Ethnicity, Muslim, Women.

Others discussed how their religion gave them comfort in times of tragedy and that the rituals around death *“were just incredibly supportive”* (FG8 Jewish Orthodox, Mixed Gender). Moreover, there was an understanding that everyone interprets and practices their religion differently and that religion is not always *“black and white*, *cast in stone”* (FG5 Bangladeshi Muslim, Women). These factors influenced discussions around the different types of autopsy and accounted for the variation in viewpoints that were observed.

#### Religious teaching on traditional autopsy

It was widely recognised in both the key informant interviews as well as amongst lay participants in the focus groups that according to the teachings of both Islam and Judaism, traditional autopsy was not religiously permitted because *“our custom*, *together with our Muslim cousins*, *is to bury as soon as possible”* (Spokesperson for Jewish Burial Society), “*that after the death the body should be left intact and it should not be disturbed unnecessarily”* (Imam), and the body “*has to be returned in the manner in which it arrived”* (P39, Jewish bereaved parent).

*“The ruling in Islam on post mortem*, *why we are so against it*, *is because the prophet Muhammed said that the one who cuts a dead body commits a sin*, *it’s like breaking the bones of a body which is alive*. *So for us*, *a dead body will feel that pain*, *so it’s a sin to cut them up”* FG7, Mixed Ethnicity, Muslim, Women.

The one example of where this view was not supported was in Liberal Judaism where “*there isn’t the same reluctance for post-mortems amongst the Reform communities generally… It’s completely acceptable*.*”* (Liberal Rabbi). However, it was also recognised that whilst Islam and Judaism may have very specific teachings on this topic, not everyone followed their religion to the same extent on this matter.

“*There are Jews who are very religiously observant*, *there are Jews who don’t care at all*. *Families vary enormously*.*”* Masorti Rabbi

Overall, the religious viewpoints from Muslim and Jewish participants differed significantly from those within the Christian faith where there’s no *“real set religious prohibition”* against autopsy (Anglo Catholic chaplain) and the general consensus is that *“when a person dies their soul is with God and therefore their body*, *whilst it has encapsulated that soul*, *is no longer needed”* (Church of England chaplain). Similarly, in Hinduism autopsy is not forbidden as *“the body has no spiritual significance at all*, *it’s just an instrument”* although traditionally the *“body needs to be cremated before the sunset”* (Hindu chaplain). Despite religious attitudes differing across the faith groups, it was acknowledged that regardless of religion, no parent would want their child to be cut or disfigured if it could be avoided.

*“It’d be very difficult to see your dead child with*, *you know*, *a huge cut along the head*, *and it would be painful… There’s something about body intactness that is almost sacred I think*, *whatever your faith*.*”* Roman Catholic Chaplain

#### Circumstances in which traditional autopsy is acceptable

The majority of parents stated that a traditional autopsy was not acceptable to them and that if a choice was offered they be likely to decline. Nevertheless, for both Muslims and Jews, the “*religion does recognise the fact that if you’re staying in a country*, *one has to abide by the laws of the country*.*”* (Spokesperson for Muslim Cemetery Trust) and hence it was accepted that in many cases, when an infant or child dies *“you need to have a cause of death so you have to have a post mortem”* (Jewish Burial Society). Participants from both faiths also acknowledged that according to their religions, saving a life was one of the most important acts one could do; “*the Koran says if you have saved one human being you have saved the whole humanity”* (Muslim Chaplain 2). Thus there was a strong case for allowing an autopsy *“if say for instance*, *somebody’s had repeated stillbirths or problems in pregnancy and it might help save a future baby”* (Orthodox Jewish Health Advocate). Similarly, if *“parents whose young child or baby has died and…if there is a necessity to look into that in more detail then generally*, *you know*, *I can't imagine there would be many scholars who would disagree that that is a legitimate reason*.*”* (Islamic Scholar).

#### Non-invasive autopsy and religious permissibility

All the religious and faith-based authorities who took part in this study agreed that from a religious perspective, NIA was permissible because it did not require any incisions or interference with the body; *“The Jewish Law has a very specific definition of what’s considered violating the body*, *and imaging doesn’t violate that”* (Orthodox Rabbi). This viewpoint was also shared by many of the focus group participants who commented that *“even if [someone is] very religious*, *they shouldn’t have a problem with going through this machine…You’re not harming the body*. *No cutting*, *no chemicals”* (FG10, Pakistani Muslim, Men). Most of the religious and faith-based authorities that were interviewed were aware that NIA had been used for coronial cases in adults for in some areas and were very much in favour of that approach.

*“We have been working very closely with the Jewish community ourselves with the various institutions*, *to ensure that where there is a possibility*, *a non-invasive post-mortem method should be utilised*. *So we’re very much in favour of CT scans*.*”* **Muslim Burial Society**

#### Uptake

Muslim and Jewish community members were positive towards the concept of NIA with the majority indicating they would potentially consent to the procedure.

“My initial thought would be, absolutely 100% I would go for a non-invasive, without any hesitation… you’re keeping the body intact.” **P43, Jewish bereaved participant**

*“I personally would say probably yes*, *because it’s non-invasive*, *it’s not going to take as long*, *you know*, *and the body’s not going to be cut*.*”* **FG5, Bangladeshi Muslim, Women**

Religious and faith-based authorities also acknowledged the potential for an increase in uptake of autopsy if a non-invasive option was available, noting that *“we might get a lot more families who will agree”* (Muslim Chaplain 1) and that *“there would be a much higher uptake*, *and not just amongst the Jewish community”* (Jewish Burial Society). Not only does NIA not violate any religious laws, but it was also considered to be psychologically easier for parents to accept because it was *“kinder to the baby”* (P44, Jewish bereaved parent), *“in terms of your mind*, *you’re put at rest*. *You don’t have that image of oh*, *gosh someone’s been cut up and then everything’s been taken out”* (FG6, Somali Muslim, Women). Familiarity with the MRI machine was also cited as a reason why some participants would be more likely to consent to NIA. There were, however, certain caveats raised, namely turnaround time, circumstances of the loss and accepting God’s will.

#### Turnaround time

Members from both faith groups discussed the importance of burying the body as soon as possible and that *“the actual soul of the person who’s passed away can’t come to eternal rest until their bodies have been buried”* (Orthodox Jewish Health Advocate), that prior to the burial *“you’re in this horrific limbo”* (FG8, Jewish Orthodox, Mixed Gender) and that the grieving process cannot begin until after the funeral. Most participants would be prepared to wait 24 hours, although others cited two or three days acknowledging that the particular circumstances would be a key factor; *“I would wait one or two days*. *I think where there were lots of miscarriages people would wait two to three days*.*”* (FG9, Pakistani Muslim, Women). Where the turnaround time was likely to be longer than 24 hours, some participants would seek advice from a religious leader. Although a swift burial was an important aspect of Jewish religious life, one Jewish leader commented that for coronial cases, *“the community would prefer to wait longer and have a non-invasive autopsy than a full autopsy and a quick burial”* (Jewish Burial Society).

#### Circumstances of the loss and likelihood of a diagnosis

Community members indicated that they were more likely to consent to NIA (or seek consent from a religious leader) if there had been multiple miscarriages or neonatal deaths, or where the information might “*help us concretely in the future”* (FG11, Jewish Haredi, Mixed Gender) such as if “*you might have more children”* (FG9, Pakistani Muslim, Women). Similarly, participants indicated they would be more likely to consent “*if the doctor said we have no idea [why this child died] then I would probably say yes”* (FG7, Mixed Ethnicity, Muslim, Women). Acceptability also depended on the chance that useful information would be found.

*“If the doctor said*, *‘Well*, *you know*, *it’s a 50/50 chance we might not get anything*, *then I don’t know if I would*.*”* **FG5, Bangladeshi Muslim, Women**

#### Accepting God or Allah’s will

Despite NIA being religiously permissible, some participants still commented that they would decline on the basis that it was unnecessary because it was the will of God; *“it was destined*, *his time was up…I don’t need any more answers”* (FG5, Bangladeshi Muslim, Women). This view was particularly prominent in the Jewish Haredi focus group.

*“As part of our religion*, *we’re not so interested in why did this happen*, *because we look at things that happen maybe with a higher purpose…the reason and the causes doesn’t really make a difference to us because*, *for us*, *if it was meant to happen*.*”* **FG 11, Jewish Haredi, Mixed Gender**

#### Placental examination

Most participants did not have any concerns with placental examination in cases of stillbirth, and were unaware of any religious observances associated with handling the placenta. A few participants commented that in cases of stillbirth *“if the placenta is connected it gets buried*.*”* (FG11, Jewish Haredi, Mixed Gender).

#### Minimally invasive autopsy and religious permissibility

It was acknowledged by both Muslim and Jewish focus group participants that strictly speaking, MIA was not religiously acceptable; *“you’re still making an incision…you’re still taking samples”* (FG8, Jewish Orthodox, Mixed Gender). *“In the Islamic perspective*, *it’s still forbidden because of the cutting*, *whether it’s a big cut or a small cut*.*”* (FG7, Mixed Ethnicity, Muslim, Women). This viewpoint was supported by religious and faith-based authorities who cited that “*it still constitutes violation of the body”* (Orthodox Rabbi) and *“from a Muslim perspective it’s not acceptable”* (Muslim Burial Society). Nevertheless, the majority acknowledged that MIA was more acceptable than a full PM; *“while it’s still forbidden it’s less forbidden”*, and *“if there was no alternative one would definitely choose the latter [MIA over full autopsy]”* (Orthodox Rabbi). Some faith-based authorities also commented that it could be religiously justified *“if parents have had more than one stillbirth or neonatal death and they are very concerned”* (Muslim Burial Society) or *“if it’s important for us to discover what happened*, *this might help us save future lives or it may be relevant to your other children”* (Masorti Rabbi) highlighting that like a traditional autopsy there are times when it could be argued that MIA is religiously permissible.

#### Uptake

Despite acknowledgment that from a religious perspective MIA was questionable, around half of focus group participants thought that this approach was acceptable, recognising that *“it’s only a small cut”* (FG4, Mixed Ethnicity, Muslim, Mixed Gender) and *“it would be a much more palatable offer than full autopsy”* (FG8, Jewish Orthodox, Mixed Gender). As with NIA, participants’ comments suggested that they would be more likely to consent to MIA if there had been multiple unexplained losses. Some stated that they would first opt for NIA but *“If there is still confusion*, *then go for minimally invasive autopsy”* (FG10, Pakistani Muslim, Men). Certain comments alluded to the fact that psychologically, MIA was preferable to a full autopsy because it was more *“respectful”* (Muslim Chaplain 1) to the body, *“it sounds nicer*, *the child would look the same afterwards”* (FG8, Jewish Orthodox, Mixed Gender) and *“psychologically it makes you think it’s OK*, *it’s just a tiny cut”* (FG7, Mixed Ethnicity, Muslim, Women). One Muslim woman was reassured that parents would potentially be able to *“direct it [the laparoscopic equipment] to where you want… they’re not going to fiddle around and take other things”* (FG6, Somali Muslim, Women). Some would first want to discuss the procedure with a religious leader before consenting. Others cited that although it was preferable to a full autopsy, they would still decline because *“the principle of cutting up*, *it still remains the same for me”* (FG10, Pakistani Muslim, Men). This viewpoint was particularly pronounced for members of the Jewish Haredi community, all of whom said they would not consent to MIA unless it was required by law.

*“Unless it’s requested by a coroner then the answer would be no*.*”* **FG11, Jewish Haredi, Mixed Gender**

#### Preference for the ‘gold standard’

A small minority of focus group participants commented that they would still choose a full autopsy over NIA or MIA as it was about *“finding answers”* (FG9, Pakistani Muslim, Women) and they would “*want to go with what has the highest success rate”* (FG8, Jewish Orthodox, Mixed Gender).

#### Recommendations for improving uptake of less invasive autopsy

Suggestions for raising awareness and improving uptake of LIA related to four themes; knowledge and awareness within the community, advice and support, challenging the status quo, and terminology (Table 3).

**Table 3:** Recommendations for improving uptake of less invasive autopsy (LIA) within the Muslim and Jewish community.

**Knowledge and awareness in the community**
• Improving awareness of the value of autopsy amongst community members.
• Educating religious leaders about LIA
• Educating the community through educational sermons at mosques/synagogues
• Hosting a conference on LIA for religious and faith based authorities.
• Raising awareness of LIA through social media platforms.
• Citing the views of religious authorities in any written or online information about LIA.
**Advice and support**
• Having hospital chaplains from the Muslim and Jewish community with knowledge about LIA and who can advise and support families.
• Training of midwives, doula’s and GP’s who work within the Muslim and Jewish community about LIA.
• Training and awareness for health professionals who might speak with parents following a loss to understand Muslim / Jewish laws and customs relating to autopsy.
**Challenging the ‘status quo’**
• Moving away from the idea that all autopsy is forbidden.
• Reducing stigma associated with autopsy amongst the Muslim and Jewish community.
**Terminology**
• Using words such as ‘imaging’, ‘scanning’, ‘MRI’ etc when describing NIA.
• Using words such as ‘keyhole surgery’ when describing MIA.
• Acknowledging that the word ‘autopsy’ or ‘post mortem’ is likely to have a negative impact and being mindful of when and how it is used.

Note: LIA = less invasive autopsy, NIA = non-invasive autopsy, MIA = minimally invasive autopsy

## Discussion

In this exploration of the acceptability of less invasive autopsy we have shown that there are no religious objections to NIA and that it is far more acceptable to Muslim and Jewish parents in the UK than a traditional autopsy which most would decline. Whilst MIA is still viewed as invasive and therefore not strictly religiously permissible, it is less objectionable than a traditional autopsy, particularly in circumstances when investigation is required (such as a Coronial procedure), and would be acceptable to a significant number of Muslim and Jewish parents if the information could help prevent losses in future pregnancies.

The findings from this study share some similarities with other authors’ findings on faith based beliefs on autopsy. Gurley et al. explored attitudes towards MIA (needle biopsy) of family members of persons who died during an outbreak of Nipah virus in Bangladesh as well as their community and religious leaders.[19] Concurrent with our study, they found that traditional autopsy was unacceptable because cutting the body is prohibited in Islam and because many respondents believed the deceased could still feel physical pain. As with our study, participants in that study believed the MIA would be more acceptable because there were no visible cuts to the body and because no organs would be removed. However, as with our study, respondents agreed that MIA would only be acceptable if there was a compelling reason to conduct it such as preventing future deaths, if religious and social customs surrounding burial were adhered to, and after seeking out the advice of religious and community leaders. Concerns around delaying the funeral were also identified by Maixenchs et al. in their study on the acceptability of MIA which was conducted in Africa and South Asia.[25] They identified a potential barrier to uptake of MIA being that death was perceived as “God’s will” and “destiny”, a finding in line with our theme of “accepting God or Allah’s will”.

Interestingly, unlike our study, acceptability of NIA was found to be low in a study conducted in Israel (which has a predominantly Jewish population) to examine the extent to which NIA has been accepted as an adjuvant or partial replacement to traditional autopsy.[37] The authors of that study found that there was no significant difference between the percentage of cases autopsied after the introduction of NIA and those before the introduction of NIA for babies/children under 18 years of age; 56% consented to standard autopsy prior to NIA, 54% after NIA and 7% consented to NIA only. The authors suggest that lack of familiarity with the procedure and mistrust of those performing the procedure might account for this low uptake. As with our study, the authors highlight the importance of ensuring religious and cultural leaders are aware of the advantages and religious permissibility of NIA and support its use.

In most focus groups and parent interviews, a diverse range of views and attitudes were expressed relating to acceptability of the three methods of autopsy, irrespective of religious group (for the Muslim participants), gender, or previous experience of loss. The one group where this differed was the Jewish Haredi focus group where all the participants identified themselves as being ‘very religious’ and attitudes towards the different types of autopsy were homogeneous. Only non-invasive autopsy was seen as acceptable in specific circumstances, with consent from a Rabbi, with all other types of autopsy considered unacceptable when not required by HM Coroner. This is likely to reflect the strict observance to Jewish law within this community and common viewpoint that the loss of a baby or child is the will of God and as such does not require further explanation (a view also expressed by a number of Muslim participants). Seeking advice from a Rabbi when faced with important life decisions is common amongst this religious group and is rooted in the principle of *da’as torah* (Torah knowledge), which mandates that one seek Torah-based guidance from a rabbinic authority on all matters of life [38].

Most religious and faith-based authorities who took part in this study were aware of the use of NIA for adult coronial cases, and valued this option in those circumstances where an autopsy was legally required. The use of NIA for adult coronial cases was established at the request of the Jewish community in Manchester in 1997 [39] and has gained increasing awareness amongst the Muslim and Jewish community in recent years [40–42]. In most of the adult cases, however, the cost of the service is covered by the community themselves [43] at a cost of around £500-£900 [44]. In 2013, the new Coroners and Justice Act recognised the importance of religious requirements relating to autopsy examination and stated that “where possible, coroners will take account of your religious and cultural needs in deciding whether to order a post-mortem examination and the type of examination to be performed” [45]. Moreover, in 2015 a high court judge backed the religious right of Jews and Muslims to ask for NIA and that it must be considered by the coroner if there was a “reasonable possibility” that it could establish the cause of death [42]. Given that NIA has been allowed within both communities for coronial cases, it is unsurprising that extending its use for non-coronial cases in childhood was perceived to be acceptable.

The findings from this study suggest that the availability of less invasive autopsy may result in an increase in uptake from members of the Muslim and Jewish community in the UK, although this is likely to require a quick turnaround to enable burial, preferably within 24 hours. Implementing such a service raises a number of practical and ethical challenges. Providing a 24/7 service would requires capital investment, training and support of healthcare management to provide dedicated autopsy imaging facilities and personnel including pathologists, radiographers and anatomical pathology technologists. These implementation challenges have been identified in previous research with health professionals and HM Coroners [46]. Moreover, important questions around prioritising access for Muslim and Jewish parents at the expense of other parents having to wait longer who may also prefer a less invasive approach need addressing. Further thought will need to be given as to how less invasive autopsy might be implemented into clinical practice in a way that is sensitive to the preferences and needs of all parents. In addition, as part of any possible future implementation study, impact of timing of release and burial in relation to examination type should be assessed.

Participants identified a number of practical recommendations for the successful implementation of less invasive autopsy amongst the Muslim and Jewish communities, largely related to raising awareness amongst the community as well as religious leaders and health professionals. Pathologists, radiologists and other professionals with experience of less invasive autopsy will need to play a key role in terms of training and community outreach. One recommendation related to the importance of training health professionals about Muslim and Jewish laws and customs concerning autopsy, particularly since many health professionals lack confidence in communicating across cultural groups different to their own [47]. However, as participants had varying attitudes towards the acceptability of different types of autopsy, health professionals must avoid cultural assumptions or stereotyping and focus on facilitating individualised care.

Less invasive autopsy was preferable to full autopsy not just for religious reasons but also because it was perceived to be ‘kinder’ and less traumatic to both parent and child. These viewpoints align with those that have been observed amongst the general parent population, where dislike of the invasiveness of the procedure and the desire to protect one’s baby or child from harm have been identified as key barriers to autopsy [7]. As such, it is highly likely that many non-Muslim and non-Jewish parents will find less invasive autopsy preferable to a traditional autopsy. Further research with bereaved parents across a wider range of cultural, ethnic and religious groups is therefore required.

### Strengths and limitations of the study

To our knowledge, this is the first study to explore in-depth the acceptability of less invasive autopsy amongst members of the Muslim and Jewish community in the UK. Even though the study was conducted in the UK, the findings may be of relevance to other countries with significant Muslim and/or Jewish populations. A key strength of the study lies in the high response rate from the religious and faith-based authorities (thus reducing the risk of non-response bias), the range of religious and faith-based authorities that were included as well as the inclusion of community members from different religious and ethnic backgrounds and locations, and with a range of experiences of loss, to ensure a wide range of viewpoints. Furthermore, we validated the findings through member checking with key informants to increase rigour. Whilst focus groups offer advantages associated with insights into attitudes and opinions that group interactions can enable, a challenge is the possibility of group dynamics promoting uniformity of views. We tried to address this by emphasising our interest in different perspectives at the start of each focus group. A further limitation is that in some cases community facilitators / key informants supporting recruitment into focus groups approached community members drawn from an established group of contacts and in some cases focus group participants knew each other. This may have impacted the diversity of opinions expressed in the group. However, given the range of opinions expressed in most group discussion, this is unlikely to have influenced our conclusions. Only six Muslim and six Jewish bereaved parents completed the questionnaire and of those only three Jewish parents took part in an interview (25% participation rate); those who did take part may have had particularly strong views on the subject. Nevertheless, nearly 60% (45 out of 76) of focus group participants indicated they had experienced some form of loss and 13 had been approached about an autopsy suggesting the subject matter was pertinent to a significant proportion of participants. We provided a generalised description of MIA to study participants involving a small incision to the stomach. It is possible that additional/alternative approaches may be developed which involve more puncture sites (e.g. up the nasal cavity or at the base of the brain) which will require comparative evaluation with parents to assess acceptability. Finally, the discussion around acceptability and likely uptake of NIA and MIA with the parents was hypothetical and may not reflect decisions people make in real life. Further research to assess actual uptake is required if less invasive autopsy becomes routinely offered in clinical practice.

## Conclusion

Less invasive autopsy appears to be more acceptable to the Muslim and Jewish community in the UK and has the potential to increase uptake in these religious groups, particularly if turnaround times can be minimised and awareness raised amongst community members. Our findings are likely to be useful for health professionals and decision makers who direct future clinical practice in this area and may be of relevance to other countries with significant Muslim and /or Jewish populations. Further work with bereaved parents from other cultural, religious and ethnic groups as well as quantitative data to provide more accurate estimates of potential uptake is required.

## Supporting information

S1 Appendix Key informant interview questions. (DOCX)

S2 Appendix Focus group questions. (DOCX)

S3 Appendix Parent interview questions. (DOCX)

S4 Appendix Description of NIA and MIA given to participants. (DOCX)
