# Supplementary material for: “We might get a lot more families who will agree”: Muslim and Jewish perspectives on less invasive perinatal and paediatric autopsy
Source: PLoS One. 2018 Aug 9;13(8):e0202023. doi: 10.1371/journal.pone.0202023 (PMC6085003; doi:10.1371/journal.pone.0202023)
Supplement: S1 Appendix — (DOCX) [file pone.0202023.s001.docx]

# S1 Appendix: Key informant interview questions

Personalising examination after death to improve experience for bereaved parents

**Key informant interviews**

1. Could you start by telling me a little bit about your religion’s view towards PM?
   1. Religious v cultural attitudes
   2. Differences amongst different religious sects

***Describe standard autopsy***

1. In your experience, have you met or worked with any parents that have lost a child/pregnancy unexpectedly and have been approached by the hospital about an autopsy?
   1. Respecting confidentiality, can you remember what their experience was and whether overall it was positive or negative?
   2. What were their reasons for accepting/declining?
   3. What questions do they ask about PM?
   4. What response do you give?
2. Could you describe how your particular religious/cultural community views autopsy?
   1. Prompt: Are there any religious/cultural reasons why it is acceptable/unacceptable? Removal of organs before returning them to body? Cutting body?
3. How do you think parents in your religion/community might feel about standard autopsy?
4. Would families from your community be treated any differently by the community if they consented or declined to standard autopsy?

***Describe non-invasive autopsy with MRI***

1. How do you think parents in your community might feel about this new method of autopsy?
2. Are there any religious/cultural reasons why this new method would be acceptable or unacceptable?
3. Do you think it would be acceptable to parents who currently decline standard autopsy?

***Describe minimally invasive autopsy with MRI and tissue sampling***

1. How do you think parents in your community might feel about this new method of investigation after death I described?
2. Are there any religious/cultural reasons that you know of why this new method would be acceptable or unacceptable?
3. Do you think it would be acceptable to parents who currently decline standard autopsy?
4. Do you have any experience of discussing MIA/NIA with bereaved parents? If YES what has been their reaction?
5. Out of all the different options we have discussed, which do you think would be preferable to people from your community?
   1. Probe: If the new methods of autopsy that I have described are both unacceptable, is there any way that autopsy could be delivered in a way that IS acceptable to your community?
6. What recommendations do you have for improving uptake of less invasive autopsy within your community?
7. Do you have any further thoughts on any of the issues we have discussed today that you think are important to mention?
