# Supplementary material for: “We might get a lot more families who will agree”: Muslim and Jewish perspectives on less invasive perinatal and paediatric autopsy
Source: PLoS One. 2018 Aug 9;13(8):e0202023. doi: 10.1371/journal.pone.0202023 (PMC6085003; doi:10.1371/journal.pone.0202023)
Supplement: S2 Appendix — (DOCX) [file pone.0202023.s002.docx]

**S2 Appendix: Focus group questions**

**Personalising examination after death to improve experience for bereaved parents**

**Focus group topic guide**

# Introduction

- Welcome everyone to the group
- Explain reason for being here today
- Doesn’t matter if they don’t know anything about post-mortem or have never had any experience of having to make a decision about PM, your views and experiences are equally valid
- Some of the things we will discuss today will be very personal. There are no right or wrong answers and I hope you will feel free to express your views and respect the views of everyone else
- I would also ask you to keep all comments made during the focus group confidential and not discuss what happened during the focus group outside the meeting.
- Using a tape recorder to record our conversation because it is difficult for me to write down everything you say and we wish to write up a report of this study to publish in academic journals and a report. Your names and personal details will not be mentioned in any report.
- Please try to speak one at a time so that I can follow what is being said.

Go around room say your name

**Discussion around standard PM**

## Describe standard PM

1. What are your thoughts about standard PM?
   1. Do you think it is a useful procedure? Why/why not?
   2. Do you think it is important to offer people PM?
   3. What do you see as being the main reasons for parents of consenting to PM following the death of a child or baby?
   4. What do you see as being the main reasons for parents declining PM?
2. In your own community, how is PM viewed?
   1. Is it considered acceptable or unacceptable?
   2. What is your understanding of the religious viewpoint on PM?
      1. For what reasons is it prohibited in the Muslim/Jewish religion?
   3. In what circumstances might it be considered acceptable?
3. Who would make the decision in your family about PM?
   1. Probe: joint decision? Woman? Man? Family? Religious leader? Wider community?

**Discussion around new methods of investigation after death**

I’m now going to describe new methods of investigation after death that have recently been developed and then ask you some questions about what you think about them.

## Describe non-invasive PM using MRI

1. What are your thoughts about this non-invasive method of PM?
   1. Advantages?
   2. Disadvantages?
2. Do you think this method is acceptable or unacceptable?
   1. Would it be considered acceptable or unacceptable from a Muslim/Jewish perspective?
   2. Are there other reasons why it might be acceptable or unacceptable? If so, what?
   3. Would it be acceptable to people who currently decline standard PM?

## Describe minimally invasive PM with MRI and tissue sampling

1. What are your thoughts about this minimally invasive method of PM?
   1. Advantanges?
   2. Disadvantages?
2. Do you think this method is acceptable or unacceptable?
   1. Would it be considered acceptable or unacceptable from a Muslim/Jewish perspective?
   2. Are there other reasons why it might be acceptable or unacceptable? If so, what?
   3. Would it be acceptable to people who currently decline standard PM?

1. Out of all the different options we have discussed, which do you think is preferable, if any?
2. Do you think you might consent to one or more of the options discussed today?
3. Are there any ways we could make PM acceptable, particularly to people who currently decline?
   1. Probe: Are there things doctors could focus on when discussing PM?
   2. Does the religious/ethnic background of the doctor matter? Why/why not?
   3. What about the language of the information/consent form?
   4. Word ‘post mortem’ or ‘autopsy’ or something else?

**Summary**

The intention tonight was to talk about how people in the community views PM. We’ve talked about reasons why people might accept standard PM, and the new methods available and whether they might be more acceptable. We’ve also talked about how doctors might improve the way they discuss this sensitive topic with patients. Have we missed anything? Is there anything else you’d like to add before we finish?

Thank you very much for your time. We are very grateful for all you contributed, we realise this can be a very emotional and delicate issue.
