# Supplementary material for: “We might get a lot more families who will agree”: Muslim and Jewish perspectives on less invasive perinatal and paediatric autopsy
Source: PLoS One. 2018 Aug 9;13(8):e0202023. doi: 10.1371/journal.pone.0202023 (PMC6085003; doi:10.1371/journal.pone.0202023)
Supplement: S3 Appendix — (DOCX) [file pone.0202023.s003.docx]

S3 Appendix: Parent interview questions

Personalising examination after death to improve experience for bereaved parents

**Introduction**

- Thank participant for taking part in the interview
- Explain that reason for this research is to understand why a large proportion of people currently decline autopsy and whether new methods of autopsy might be more acceptable.
- Recognise that this is a very sensitive subject matter and I can stop the interview at any time if it gets upsetting. Express sympathy of their loss.
- Can also choose to withdraw from study and delete the audio-recording if you would like.

**Feedback on questionnaire**

1. Could I start by just asking you if you had any feedback on the questionnaire?
2. We know this is a very sensitive subject. What were your thoughts about being asked to take part in a questionnaire about this topic?

**Own experience of PM**

1. One of the key aims of this interview is to discuss your views about new methods of autopsy, but I wonder if before we speak about that you could tell me about your own experience of being approached about an autopsy examination.
   1. Prompt: were you expecting to be approached about it?
   2. Who approached you?
   3. What did they discuss?
   4. What type of autopsy were you offered (standard, MIA, NIA, compulsory)?
   5. Were you given any written/printed information?
   6. How much time did you have to think about it?
2. Did you agree or decline autopsy? Could you describe your reasons for accepting/declining autopsy?
   1. Probe: religious or moral reasons for accepting/declining
3. What are your thoughts about how the topic was discussed? Do you have any suggestions for how that discussion could be improved?
   1. How good/clear was the consent form and other written information?
   2. Timing of discussion
   3. Where discussion took place
   4. The way the information was communicated
   5. Support in decision-making
4. (For those who had PM) Did you get the information you needed from the PM?
   1. How long did it take to get the results?
   2. Were they helpful? In what way?
   3. After the PM, did you see the baby/child? What was that like?
5. Reflecting back on your experience, how do you feel now about agreeing/declining PM?
   1. Has there been any long term impact .g. psychological
   2. Are these related to procedure itself .e.g invasiveness?
   3. Impact on having further children
   4. Any regrets about accepting / declining?

**Introduce non-invasive autopsy with MRI**

1. What are your thoughts about this new method?
   1. Prompt: is it preferable or not to the standard method?
   2. What are the advantages?
   3. What are the disadvantages?
   4. Views about placenta being taken away for examination?
   5. Views about baby being in the MRI scanner?
2. Would MIA with MRI be acceptable to you? Why/why not?

**Introduce MIA with MRI and tissue sampling**

1. What are your thoughts on this method?
   1. Prompt: is it preferable or not to the standard method?
   2. Is it preferable to the MIA method?
   3. What are the advantages?
   4. What are the disadvantages?
   5. Views on size of cut
2. Would MIA with tissue sampling be acceptable to you?
3. If you had been offered all 3 options, which would you have chosen? Why? Why not the other methods?

**General**

1. Do you have any other thoughts on these new methods of autopsy, or autopsy in general that you would like to share?
2. Do you have any thoughts about the terminology health professionals should use when discussing investigation after the loss of a baby/child?
   1. Probe: Is the term ‘investigation after death’ preferable to post mortem or autopsy?
   2. What about the terms I’ve been using for these new methods such as minimally invasive autopsy and non-invasive autopsy?
   3. Are there any other suggestions you have for the terminology we could use?
3. *For parents for whom compulsory autopsy was required by HM coroner.*
   1. At present compulsory autopsies as required by HM coroner are conducted as standard autopsies. What would be your views on using less invasive methods of examination in these cases?
   2. Do you think a choice of methods (standard/MA/NIA) should be offered to parents in this situation? Why?
