# Supplementary material for: “We might get a lot more families who will agree”: Muslim and Jewish perspectives on less invasive perinatal and paediatric autopsy
Source: PLoS One. 2018 Aug 9;13(8):e0202023. doi: 10.1371/journal.pone.0202023 (PMC6085003; doi:10.1371/journal.pone.0202023)
Supplement: S4 Appendix — (DOCX) [file pone.0202023.s004.docx]

S4 Appendix: Description of non-invasive autopsy and minimally invasive autopsy given to interview and focus group participants

***Standard autopsy:***

- A procedure like an operation in which all parts of the body can be examined in detail including the internal organs (lungs, heart etc);

- A cut is made in the chest and stomach and each organ is removed and examined;

- Depending on the circumstances a cut may also be made around the head so that the brain can be removed and examined;

- In most cases the doctor will remove a small sample of tissue from these organs to examine later under a microscope;

- The organs are then put back and the cut is closed securely.

***Non-invasive autopsy:***

- Does not require any incisions to the body;

- Is based around an examination of the body using imaging, either an MRI or CT scan, which is non-invasive;

- May also include examination of the placenta or bloods if appropriate;

- Is non-invasive and therefore no tissue is available for analysis.

***Minimally invasive autopsy:***

- Involves imaging (as above)

- Also includes examination of internal organs and tissue sampling done using a ‘keyhole surgery’ approach;

- Requires a small incision (around 10-20mm) to the stomach area;

- Following the procedure the cut is closed with stitches;

- There are very few marks left on the body compared to a full autopsy.
